# Optic nerve injury-associated blunt cerebrovascular injury: Three case reports

**DOI:** 10.1097/MD.0000000000008523

**Published:** 2017-11-10

**Authors:** Dan-Dong Li, Liu-Xun Hu, Linyuan Sima, Shang-Yu Xu, Jian Lin, Nu Zhang, Bo Yin

**Affiliations:** Department of Neurosurgery, The Second Affiliated Hospital and Yuying Childern's Hospital of Wenzhou Medical University, Wenzhou, Zhejiang, China.

**Keywords:** blunt cerebrovascular injury, blunt intracranial carotid injury, craniocervical trauma, optic nerve injury

## Abstract

**Rationale:**

Blunt cerebrovascular injury (BCVI) is a rare complication that may occur after craniocervical trauma. The current literature is limited to extracranial carotid artery injuries; however, no reports have been published on blunt intracranial carotid injury (BICI), especially those associated with optic nerve injury.

**Patient concerns:**

Here we report on 3 BICI cases that demonstrated optic nerve injuries after craniofacial injuries. All 3 patients showed post-trauma vision loss on the injured side.

**Diagnoses:**

Optical canal fractures can be found in these patients, and carotid sulcus was compressed by the fragments. Computed tomography angiography (CTA) and digital subtraction angiography (DSA) were performed in all 3 patients.

**Interventions:**

Case 1 was given no further treatment, except for symptomatic support and rehabilitation therapy. Case 2 was treated with antiplatelet therapy for 3 days, and then a stent was inserted in the injured internal carotid. Case 3 received antiplatelet therapy and a internal carotid compression test was performed simultaneously for 2 weeks, then the injured internal carotid was completely blocked.

**Outcomes:**

Case 1 developed cerebral infarction that resulted in unilateral hemiplegia. Due to timely treatment, the remaining 2 patients had a better prognosis.

**Lessons:**

CTA should be performed primarily to exclude vascular injury and for CTA-positive patients, a further DSA should be performed to investigate pathological changes and for a definitive diagnosis. At last, the current therapeutic protocols for BCVI are not entirely applicable to intracranial vascular injury, and appropriate protocols for the treatment of BICI should be selected based on the combination of test results and the actual condition of the patient.

## 1 Introduction

Blunt cerebrovascular injury (BCVI) occurs in 0.08% to 2.7% of all blunt traumatic injuries. About 39% to 60% of patients with BCVI develop ischemia, and among those, 30% to 80% of patients die.^[1–5]^ Because the clinical signs and symptoms of BCVI are frequently absent at the stage when injuries can be treated to prevent neurologic complications, the diagnosis of BCVI is a challenging task in recent trauma care. Various screening criteria have been advocated, and comprehensive screening for BCVI has shown to increase the detection of BCVI; however, up to 20% to 30% of BCVI could be missed using this set of screening criteria^[4,6,7]^; therefore, optimal screening criteria have yet to be identified. Current screening criteria mainly focus on screening the extracranial carotid artery or vertebral artery, and only few articles can be found that focus on diagnostic and therapeutic approaches for blunt intracranial carotid injury (BICI). In fact, numerous injury-prone anatomical structures surround the intracranial carotid artery, especially the optic nerve and its adjacent structures such as the optic canal, and ligaments are directly connected to the carotid artery. The blunt trauma-injured optic nerve may also injure the ipsilateral carotid artery. Moreover, the therapeutic principles between intra and extracranial carotid injury are different. The main goals of treating BICI patients are to prevent hemorrhage and ischemic infarction, whereas for cervical BCVI patients, treatment is more focused on preventing cerebral infarction. Therefore, the current screening protocols and therapies are need to be further discussed.

In this article, we report on 3 cases with optical nerve injury-associated BICI after head trauma. All patients were hospitalized for optic nerve injury and suffered from stroke during the preoperative preparation period for optic canal decompression. In addition, we describe a category of injuries associated with BICI that should be taken into account.

## 2 Case materials

### 2.1 Case 1

**Figure 1:**
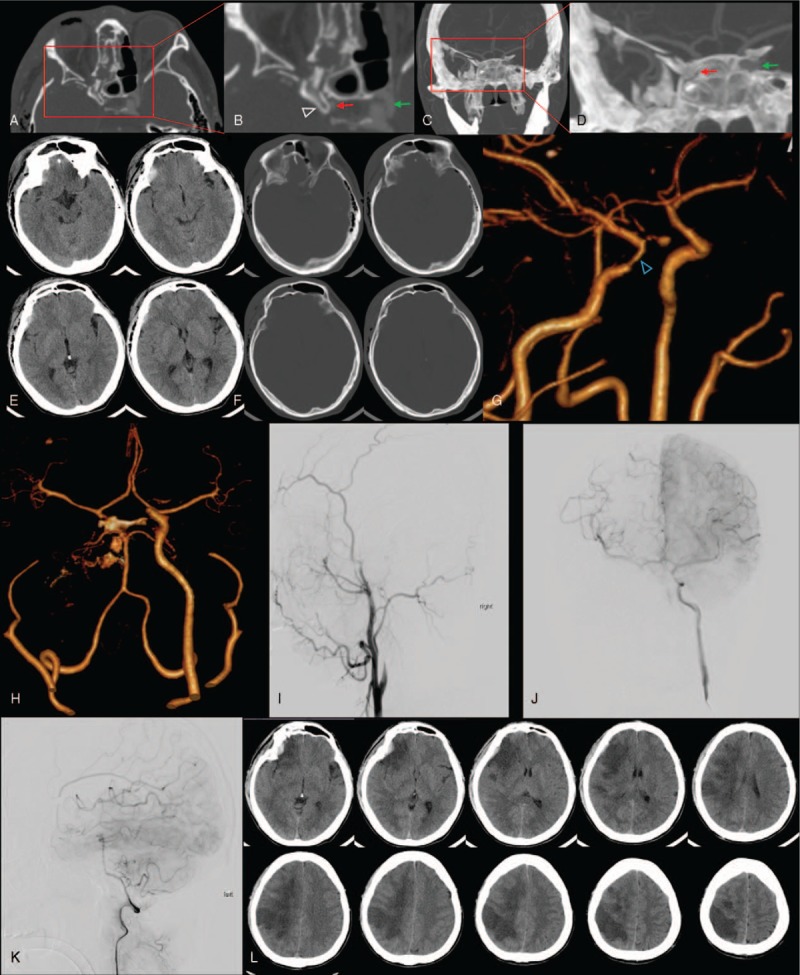
Figures from case 1. (A) The orbit computed tomography (CT) and (B) the enlarged figure from red square. The white hollow arrow indicates anterior clinoid process (ACP), red arrow indicates right internal carotid artery (ICA), and green arrow indicates left ICA. The right ICA is extruded by ACP. (C) The coronal scan and the (D) enlarged figure from rad square. (E) and (F) The initial head CT scan. (G) The 3D reconstructed image of head CT angiography (CTA). The blue hollow arrow refers to arterial stenosis. (H) The second CTA. The right ICA disappeared. The digital subtraction angiography (DSA) figures of right ICA (I), left ICA (J), and left vertebral artery (K). (L) The head CT scan showed cerebral infarction.

A 36-year-old male was admitted to the emergency room of our hospital with a primary complaint of blindness of the right eye for 2 days after head injury. The patient's head was hit by a heavy object 2 days before, which resulted in pain and blindness of the right eye. The patient was transferred to our hospital after treatment for a duration of 2 days in another hospital which was unsuccessful. General examination indicated that the patient was alert, but bruises and swelling were present around the right eyelid. The right eye showed no light perception, and the pupil was dilated and fixed with no reflex to direct and indirect light signals. The left eye appeared normal. No otorrhea or rhinorrhea of the cerebrospinal fluid was observed. No other symptoms were found during neurological examination. A computed tomography (CT) scan of the head and orbit showed a fractured right frontotemporal bone with a small right frontal epidural hematoma in addition to multiple fractures of the right frontal temporal bone, right orbital lateral wall, right anterior and lateral maxillary sinus, right zygomatic arch, right alisphenoid and sphenoid body, left nasal bone, and nasal septum (Fig. 1). A head computed tomography angiography (CTA) scan demonstrated that the walls of the left carotid had a rough appearance, combined with ∼30% local stenosis. As an antiplatelet therapy, Bayaspirin was given orally at a dose of 100 mg/d. The next morning, approximately 12 hours after the CTA scan, when the patient woke up, he was found unconscious. The patient's left upper and lower limbs muscle strength was reduced to 3. A head CT scan revealed an infarction of the right brain; moreover, a head CTA scan demonstrated no visualization of the right carotid artery. The digital subtraction angiography (DSA) showed no visualization of the right internal carotid artery (ICA); however, blood circulation was compensated by the left ICA. During angiography, blood vessels in the right hemisphere demonstrated slow image development. The posterior cerebral circulation could partially compensate for the defect; however, this angiography development was also on the slow side. Given the infarction and compensatory circulation, this patient was given no further treatment, except for symptomatic support and rehabilitation therapy. Three months later, the patient was given a modified Rankin Scale (mRS) score of 3.

### 2.2 Case 2

**Figure 2:**
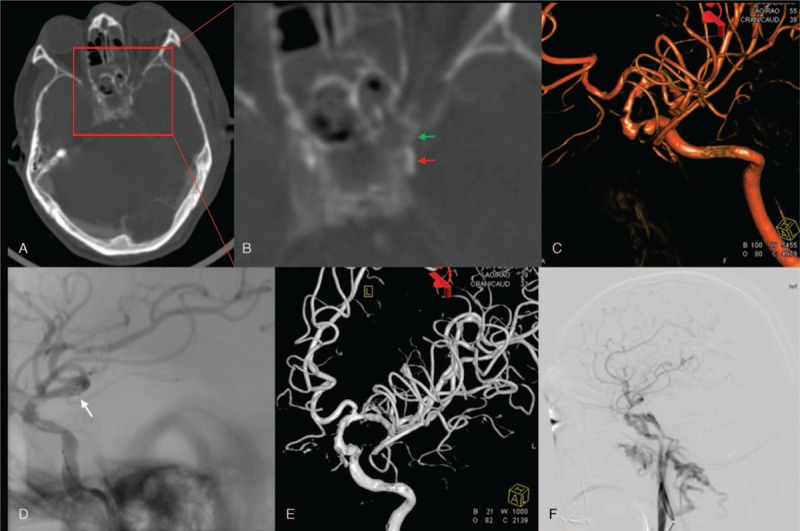
Figures from case 2. (A) The orbit computed tomography (CT) scan and (B) the enlarged figure from red square. The red arrow indicates left internal carotid artery (ICA) and the green arrow indicates fracture flat. (C) The 3-dimensional (3D) reconstructed image of head computed tomography angiography (CTA). (D) The angiography before stent release. The white arrow referes to the distal markers of stent. (E) The 3D reconstructed image after stent release. (F) The DSA follow-up after 3 months, left carotid-cavernous fistula was observed.

A 58-year-old male entered our hospital for optic canal decompression, and a chief complaint of losing sight in the left eye 2 days after a fall. This patient was treated in another hospital for the duration of 2 days; however, the exact treatment approach is unknown. During general examination, it was found that the patient was alert, but had bruises and swelling of the left eye socket and signs of edema at the eyelid. The left eye was fixed and did not have any light perception. The left pupil was dilated and fixed with no reflex to direct light signals and a slow reaction to indirect light. Neurological examination indicated that no other symptoms were present. A head and orbital CT scan revealed left temporal epidural hematoma, a small amount of subarachnoid hemorrhage, and multiple fractures of the left temporal frontal bone, maxillofacial bones (Fig. 2). A head CTA scan demonstrated stenosis of the left ICA. DSA examination demonstrated left ICA dissection and the presence of ∼45% local stenosis. Antiplatelet therapy (Bayaspirin 100 mg/d + Plavix 75 mg/d) was given, and a stent (Solitaire AB 6–30) was inserted in the left ICA 3 days later; the patient was discharged 3 days later and did not show any deficiency in neurological function. Three months after the procedure, the patient had a mRS score of 1. During DSA follow-up, left carotid-cavernous fistula was observed; however, the patient did not feel any discomfort and refused further treatment.

### 2.3 Case 3

**Figure 3:**
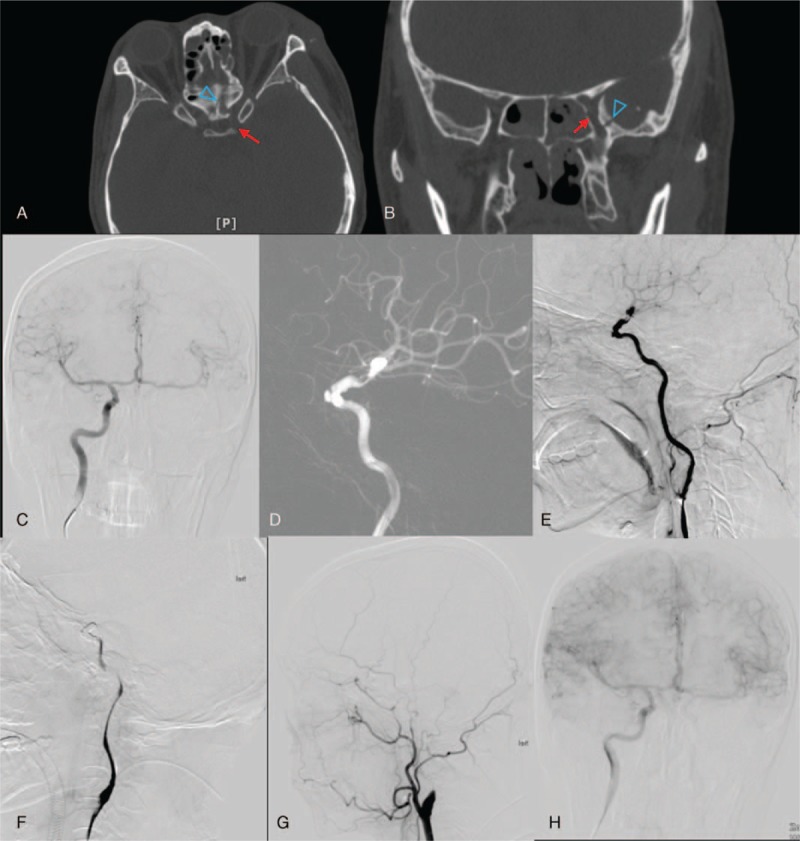
Figures from case 3. (A) and (B) The orbit computed tomography (CT) scan. The blue hollow arrow indicates fracture line and the red arrow indicates left internal carotid artery (ICA). The digital subtraction angiography (DSA) figures of right ICA (C), and left ICA (D) and (E). (F) The DSA figures after vascular occlusion. The DSA follow-up after 3 months, left ICA (G) and right ICA (H).

A 42-year-old male arrived at our hospital for optic canal decompression and complained about left eye blindness for 6 days after a traffic accident. An orbital CT scan showed multiple fractures of the maxillofacial bones with possible compression of the ICA (Fig. 3). Further DSA examination showed that the entire left ICA had thinned out. In addition, blood flow was slow, and filling defects and uneven blood vessel walls at C5, C6, and C7 segments were observed. This caused reduced image development of the ipsilateral middle cerebral artery and compression of blood vessels by bone fragments; however, no leakage of contrast medium was observed. The right ICA compensated the left anterior and middle cerebral arteries with sufficient blood supply, which resulted in synchronization of bilateral image development. The patient received antiplatelet therapy (Bayaspirin 100 mg/d and Plavix 75 mg/d), and a left ICA compression test (15–30 minutes, 2 times/d) was performed simultaneously for 2 weeks. After 2 weeks, the left ICA was completely blocked from the proximal cavernous segment to the distal posterior communicating artery segment. Three months after surgery the patient received a mRS score of 1. During follow-up, DSA indicated occlusion of the left ICA; however, blood circulation was sufficiently compensated by the right ICA.

Consents to conduct and report of this study were obtained from the Ethics Committee of Second Affiliated Hospital of Wenzhou Medical University (Wenzhou, China). The patients provided informed consents for the publication of their clinical and radiological data.

## 3 Discussion

Early diagnosis is critical for successful treatment of BCVI. Since the first published report by Verneuil et al in 1872, numerous studies have focused on identifying novel approaches to screen and identify the high-risk patient population in the asymptomatic phase for early intervention. Many screening guidelines exist (Table 1) and have been proposed by different investigators and institutions.^[6,8–10]^ Unfortunately, current guidelines are more applicable for screening injuries of extracranial blood vessels. The only 1 entry for intracranial ICA injury is the “basilar skull fracture,” which is more related to injuries of the petrous and lacerum segments of the ICA.^[3,8,9]^ No description can be found on injuries of the cavernous or clinoid segments, nor is there any report that focuses on analyzing optic nerve injury-related ICA injuries. Due to the close anatomical relation between the optic nerve, optic canal, and ICA, injuries in this area often result in ICA injury. In this report, we highlight 3 cases of ICA injury that relate to optic nerve injury and emphasize the importance of such cases during clinical practice.

**Table 1:**
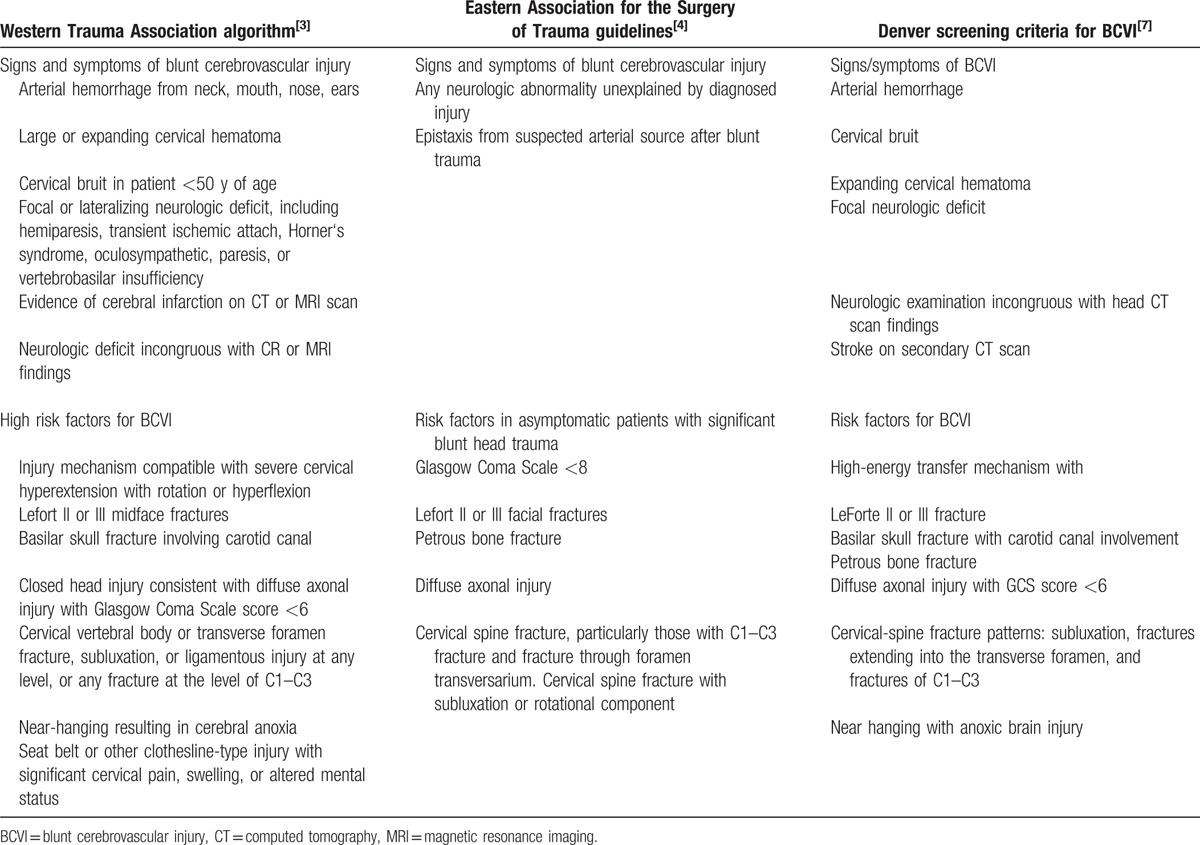
Screening criteria and risk factors for blunt cerebrovascular injury from 3 current major protocols.

The carotid sulcus is a shallow groove on the body of the sphenoid bone, which starts from the intracranial end of the carotid canal.^[11]^ After the carotid canal, the carotid sulcus turns anteriorly along the exterior margin of the sella turcica and ascends along the exterior margin of the anterior wall of the sella turcica. It then passes the posterior side of the optic strut and the interior margin of the anterior clinoid process (ACP) and into the dura matter. The posterior part of the ACP often surrounds the ICA from the lateral side, and in some cases the carotid-clinoid foramen is formed by ossification of the ligaments from the ACP and medial clinoid process (MCP) at the end of the carotid sulcus through which the ICA passes.^[12]^ The presence of this foramen increases the risk of carotid injury. The ACP is connected to the sphenoid bone by 3 bony structures, which are the roof of the optic canal that is formed by the forward connection of the basal ACP and anterior root of the lesser sphenoid wing.^[13–15]^ The bottom of the optic canal is formed by the backward connection of the basal ACP and the posterior root, and is known as the optic strut, and the lateral wall of the optic canal is formed by the interior margin of the basal ACP. These anatomic features of adjacent structures that are present along passage of the ICA suggest that fractures involving the optic canal can produce bone fragments that affect not only the optic nerve, but also the adjacent ICA. This represents the fundamental osseous anatomy for ICA injury that is associated with optic nerve injury.

The top of the cavernous sinus comprises 2 layers of dura mater that separately wrap around the ACP. The outer dural layer wraps around the superior surface of the ACP and the inner dural layer covers the inferior surface of the ACP. Both structures extend further to the sphenoid bone.^[16]^ The clinoid segment (C5) of the ICA travels between the 2 dural layers, and the outer dura mater extends inwardly and fuses with the adventitia of the ICA to form the distal dural ring.^[14,16]^ This distal dural ring is an anatomic landmark of the ICA entering the dura mater. The dural layer continues to cover the sphenoidal platform and sella turcica to form the falciform ligament and the dural sheath of the optic nerve.^[17]^ The ICA, the optic nerve, the sphenoidal platform, and the sella turcica are tightly connected via the dura mater. When these structures are shifted by an external force, the strong impact can be transmitted through the dura mater to adjacent structures. Given that the distal dural ring is fused with the adventitia of the ICA, a violent stretch on the distal dural ring may cause damage on the adventitia of the ICA. Although the proximal dural ring, unlike the distal dural ring, is not fused with the adventitia of the ICA and there is a gap between the 2, the proximal dura ring can continue to wrap the artery upward.^[16]^ Therefore, the ICA is encircled in a dural sleeve, which is a continuation of the proximal dural ring. The above represents the fundamental dural anatomy for ICA injury associated with optic nerve injury.

Since the ICA is a muscular artery, the tunica media, which has relatively limited elasticity, is mainly composed of smooth muscle cells. The tunica media of the epidural section of the ICA has 2 elastic layers—the inner elastic layer and the outer elastic layer; however, the inner-dural section of the ICA lacks the outer elastic layer.^[18,19]^ Previous studies have shown that the outer elastic layer of the ICA is still intact in the lacerum segment (C3). It gradually becomes thinner from the proximal to the distal sections of the cavernous segment (C4) and completely disappears in the clinoid segment (C5).^[19]^ Although the C5 segment, which is located between the 2 dural rings, is considered epidural, the composition of the vessel wall is closer to that of inner-dural ICA, which is more prone to injury than the C3 and C4 segments. These are the intrinsic bases for ICA injury that is associated with optic nerve injury.

We believe that the following mechanisms are involved in optic nerve injury-related ICA injuries. A forceful blow can cause fractures of the optic canal and its adjacent bone structures, resulting in optic nerve contusion. Fracture-induced shift of bony structures such as the ACP, the optic strut, and the lateral wall of the sphenoid bone may further result in direct compression of the ICA. In addition, the high impact can pull the dura mater that covers the surface of the bones and transfer the impact force to the adjacent optic nerve and ICA, thereby causing additional injury. Furthermore, the unique structure of the clinoid segment is an important risk factor for ICA injury.

Currently, there is no uniform standard protocol for treating BCVI patients. In 2014, the Denver Health Medical Center published a study in which the treatment outcome of 195 BCVI patients over a 15-year period was evaluated.^[20]^ This study identified that anticoagulant therapy is effective in preventing stroke in patients with Denver grade II and III BCVI. Moreover, stent procedures not only failed to improve in vivo efficacy but also increased in costs and risk factors. Unfortunately, this study did not distinguish between intracranial and extracranial injuries. Unlike the extracranial ICA, the intracranial ICA has a relatively decent inner elastic lamina with less elastic fibers in the tunica media and a small amount of adventitial tissue; however, it does not have the outer elastic layer. Therefore, when intracranial vascular dissection occurs, sufficient supportive tissue, such as found in cervical blood vessels, cannot be provided. This results in an increased probability of, for example, dissection of the intracranial arterial adventitia and secondary hemorrhage.^[21]^ In the more severe cases, hemorrhage could occur. Furthermore, intracranial vascular injury is often accompanied with traumatic brain injury, which may complicate the decision for further treatment. The main goals of treating BICI patients are to prevent hemorrhage and ischemic infarction, whereas for cervical BCVI patients, treatment is more focused on preventing cerebral infarction. Currently, large-scale randomized controlled trials on intracranial arterial dissection have not been performed, and evidence in this area is not strong.^[21]^ In clinical practice, the treatment decision is more difficult and frequently based on empirical therapy. It is our hope that our experience and lessons learned from previous studies would serve as useful source for other clinicians.

Although in case 1 of the current study, aspirin antiplatelet therapy failed, this does not indicate that antiplatelet therapy is unsuitable for patients with BCVI. On the contrary, previous studies have shown that for Denver grade I patients who show less than 25% stenosis, noninvasive antithrombosis treatment is the most suitable treatment method.^[20]^ For patients with intracranial hematoma, the choice of treatment is more challenging. Currently, there is no decisive antithrombosis treatment protocol available, and the doses used vary depending on the patient treated. Numerous studies indicate that there is no difference between antiplatelet and anticoagulant therapy^[21,22]^; however, continuous intravenous heparin infusion can reach the plasma concentration within minutes, whereas for oral aspirin administration, it takes approximately 3.5 hours to reach the plasma concentration. In a previous study, it was recommended that continuous intravenous infusion of 15U/kg/h heparin should be used in the acute phase.^[20,22]^

Endovascular treatment of intracranial arterial dissection includes luminal reconstruction and vascular occlusion, which represent reconstructive therapy and deconstructive therapy, respectively. As the primary treatment procedure in case 2 of this report, a stent procedure was chosen because insufficient ipsilateral compensation was found after carotid compression testing. During the procedure, a Solitaire AB(6.0 × 30 mm) stent was inserted. Postoperative antiplatelet therapy using Bayaspirin (100 mg/d) and Plavix (75 mg/d) was given. The glasgow outcome scale score at the time of discharge was 5. During follow-up visit, 3 months later, carotid cavernous fistula (CCF) was demonstrated. This could be due to 1 of following reasons:

1. Original bone fragments that compressed the blood vessels were not completely removed. This resulted in compression to persist after insertion of the stent, which lead to the formation of fistula.
2. The fistula were already present, but were not notified earlier because of thrombosis or collapsed endothelium and may have reopened after insertion of the stent.
3. The blood vessel wall at fistula sites was weak and ruptured after stent insertion.

Occlusion treatment of the patient in case 3 resulted in a better outcome. The initial DSA revealed sufficient contralateral compensation and carotid compression testing was well-tolerated. However, 2 weeks after the carotid compression test, the carotid artery was occluded. At present, the choice of endovascular treatment in BICI patients is still controversial.

In all 3 cases, DSA examination showed no signs of ophthalmic artery, and vision loss had occurred for more than 2 days. There was no reason for optic canal decompression treatment; therefore, none of the patients underwent surgery. The vision loss of the affected side was permanent.

## 4 Conclusions

In the present report, we reported 3 blunt intracranial carotid injury cases that were associated optic nerve injury. We discussed the anatomic-prone injury factors, and the special screening and therapeutic protocols different from extracranial carotid injury. We believe that CTA or DSA should be performed in the craniocervical trauma patients associated with optic nerve injury to exclude intracranial carotid injury.

## Acknowledgments

The authors acknowledge the contribution of the staff on the department of neurosurgery, ophthalmology, and the emergency department for the excellent care they have provided to these patients.
